# Diffuse malignant peritoneal mesothelioma: A review

**DOI:** 10.3389/fsurg.2022.1015884

**Published:** 2023-01-06

**Authors:** Luanbiao Sun, Chenguang Li, Shuohui Gao

**Affiliations:** Gastrointestinal Colorectal and Anal Surgery, China-Japan Union Hospital, Changchun, China

**Keywords:** diffuse malignant peritoneal mesothelioma (DMPM), hyperthermic intraperitoneal chemotherapy (hipec), immunotherapy, targeted molecular therapy, signaling pathway

## Abstract

Diffuse malignant peritoneal mesothelioma (DMPM) is an unusual and life-threatening locally invasive tumor. The morbidity and mortality of the disease are associated with progressive local effects in the abdominal cavity, such as abdominal distention, painful sensations, and early saturation with reduced oral intake, which eventually lead to intestinal obstruction and cachexia. Computed tomography (CT) has been widely used as a first-line diagnostic tool for DMPM. In addition, the most sensitive immunohistochemical markers of DMPM include WT 1, D2-40, and calmodulin. This paradigm has altered with the advancements in the immunohistochemical analysis of BRCA1-Associated Protein 1 (BAP1) the lack of BAP1 expression shows the diagnosis of malignancy. DMPM is resistant to conventional chemotherapies. Therefore, the gold standard for the treatment of DMPM is the combination of cytoreductive surgery (CRS) and hyperthermic intraperitoneal chemotherapy (HIPEC). The overexpression of the phosphatidylinositol 3-kinase (PI3K)/AKT serine/threonine kinase 1 (AKT)/mammalian target of rapamycin (mTOR) signaling pathway drives the malignant phenotype of DMPM, thereby showing promising potential for the treatment of DMPM. The coordinated activities among multiple RTKs are directly involved in the biological processes of DMPM, suggesting that the combined inhibition of the PI3K and mTOR signaling pathways might be an effective measure. This treatment strategy can be easily implemented in clinical practice. However, the combined inhibition of ERBB1(HER1)/ERBB2 (HER2) and ERBB3 (HER3) requires further investigations. Thus, based on these, the discovery of novel targeted therapies might be crucial to improving the prognosis of DMPM patients.

## Introduction

Malignant peritoneal mesothelioma (MPM) is an unusual and invasive primary malignancy of the peritoneum, which is characterized by the widespread multiple meta-static nodules, originating from the peritoneum. MPM has been conventionally classified into diffuse MPM (DMPM) and border-line forming MPM, including multi-cystic PM (MCPM) and well-differentiated papillary PM (WDPPM). DMPM is a rare type of primary malignancy, originating from the mesothelial cells in the peritoneum, and is characterized by a diffused and invasive growth of the tumor along the peritoneal surface.

## Incidence and epidemiology

DMPM accounts for 7%–30% of mesotheliomas (1). Wynn and Miller first reported DMPM for the first time in 1908 (2). The global epidemiological data of DMPM varies due to differences in geographical locations, genetic susceptibilities, and exposure levels of environmental and occupational carcinogens. The United Kingdom, Australia, and New Zealand have the highest incidence rates, while Japan, Slovenia, and other central European countries have the lowest incidence rates. The median age at the time of DMPM diagnosis is earlier than other peritoneal surface malignancies (63 71 years). Males are more likely to develop pleural mesothelioma, while females are more likely to develop DMPM. Moreover, DMPM occurs in younger females more likely as compared to the DMPM, occurring in male patients. The incidence rates of DMPM in the United States are 19.4 million and 4.1 million among the male and females populations, respectively, with about 15,000 new confirmed cases each year; the median age at the time of diagnosis is 63.3 years with a latency period of about 40–50 years from asbestos exposure to disease development (3, 4). There are limited epidemiological studies conducted on DMPM in China. Zhao et al. reported that the overall incidence and mortality rates increased from 2.14 to 3.14 million and 1.24 to 2.44 million, respectively, in the asbestos-exposed population at the time of DMPM diagnosis in China from 2000 to 2013. The mean ages at the time of DMPM diagnosis were 55.2 years in the exposed population and 47.3 years in the non-exposed population (5).

## Etiology and pathogenesis

Asbestos is believed to be the most frequent carcinogen, causing pleural mesothelioma. Although it has a weak correlation, it is considered one of the high-risk factors for DMPM. Approximately, one-third of the DMPM patients have a history of previous asbestos exposure (4). The timing and duration of asbestos exposure are not directly correlated with the disease progression, suggesting that long-term asbestos exposure might not cause DMPM. On the contrary, the short-term exposure might cause a substantial tumor burden. Numerous randomized and observational studies, including the National Lung Screening Trial (NLST) and International Early Lung Cancer Action Program (IELCAP), screened asbestos-exposed workers using chest computed tomography (CT) for lung screening programs. Although there is a moderately consistent epidemiological correlation between the DMPM and asbestos exposure, no screening program or plan has been proposed for the early detection of DMPM. Therefore, researchers have recommended annual abdominal ultrasonography for individuals with a history of asbestos exposure to improve early detection (6). Other physicochemical carcinogens include gross zeolite, xylene, mica, and talcum powder. The other physical factors associated with DMPM include chronic peritonitis and therapeutic radiation. In addition, DMPM is also associated with genetic susceptibility and simulated jejunum 40 (7).

## Clinical presentation

Most DMPM cases are asymptomatic or non-specific in their early stages. However, DMPM has an insidious onset and is diagnosed in the middle to late stages with a median time from the onset of symptoms to diagnosis of approximately four months. The diversified clinical presentations mainly depend on the degree of intra-abdominal spread. The most common symptoms include abdominal distention (41%–86%) and abdominal pain (31%–87%). Other clinical manifestations include weight loss (32%), abdominal masses (30%), fever (22%), diarrhea (17%), vomiting (15%), and new hernias (12%). In addition, about 8% of the cases are incidentally diagnosed. The typical growth of DMPM is characterized by an extensive growth along the peritoneal surface with little involvement of the extra-abdominal organs. The enlargement of the local lymph node might obstruct the superior vena cava or compress the vital organs, thereby showing the corresponding signs and symptoms. In some patients, the acute abdominal disease is the primary clinical manifestation, such as malignant intestinal obstruction or gastrointestinal perforation. During its progression, DMPM might also be accompanied by paraneoplastic syndromes, such as fever, thrombocytopenia, malignancy-associated thrombosis, hypoglycemia, Coombs-positive hemolytic anemia, and nephrotic syndrome.

## Staging

**Figure 1:**
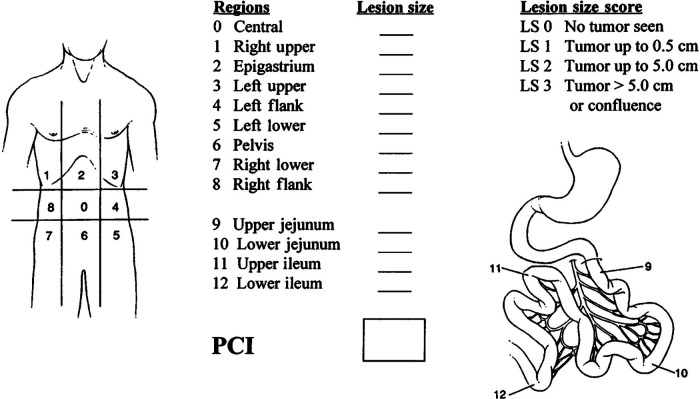
Peritoneal cancer Index (PCI) scoring system (9). Lesion size (LS) should be considered. The absence of malignant deposits is indicated by an LS-0 score: the tumor nodules less than 0.5 cm are indicated by an LS-1 score (the number of tumor nodules is not scored; only the size of the largest nodule is calculated); tumor nodules between 0.5 cm and 5.0 cm are indicated by an LS-2 score; and tumor nodules of more than 5.0 cm in either direction are indicated by LS-3 score. The confluence of stratification of tumors also indicates a score of LS-3 in the abdominal or pelvic areas. Each of the 13 areas received an LS score. The patient's peritoneal cancer index was calculated as the average lesion size score for each of the 13 abdominal-pelvic areas. The highest score was 39 or 13*3.

Due to the inconsistent occurrence of lymph nodes and spread of metastasis, DMPM does not fit into the typical Tumor-Node-Metastasis (TNM) staging system for tumors. Yan et al. [2011] presented a staging system based on the degrees of peritoneal disease burden (T), intraperitoneal lymph node metastasis (N), and extraperitoneal metastasis (M) (8). The T stage was determined based on the calculation of the peritoneal carcinoma index (PCI) (Figure 1) (9). The Peritoneal Surface Oncology Group International(PSOGI) classified DMPM into three stages, including Stages I, II, and III, based on this TNM principle (Table 1).

**Table 1:** TNM staging system for diffuse malignant peritoneal mesothelioma (DMPM).

Stage	PCI	T	N	M	5-year survival rates
I	1–10	T_1_	N_0_	M_0_	87%
II	11–30	T_2–3_	N_0_	M_0_	53%
III	≥30	T_4_	N_0–1_	M_0–1_	29%
		T_1–4_	N_1_	M_0–1_	
		T_1–4_	N_0–1_	M_1_	

T refers to PCI: T_1_ (PCI 1–10), T_2_ (PCI 11–20), T_3_ (PCI 21–30), and T_4_ (PCI 30–39), which were significantly associated with the patient survival rates. N refers to lymph node status: N_0_ without lymph node metastasis and N_1_ with lymph node metastasis. M refers to extra-abdominal metastasis: M_0_ without extra-abdominal metastasis and M_1_ extra-abdominal metastasis.

## Diagnosis and pathology

### Diagnostic imaging/preoperative work-ups

#### Computed tomography (CT)

Currently, for the preoperative evaluation of DMPM, the CT scan is the preferred radiological method (10). This might be due to the feasibility, cost, short acquisition time, and relative ease of interpretation by non-trained radiologists. In addition, a CT scan can detect peritoneal diseases either malignant disease or plaques, and can identify asbestos exposure. In general, a CT scan can show mesenteric thickening, peritoneal effusion, greater omental thickening, peritoneal thickening, abdominal masses, and extra-abdominal metastases (11). Recent studies suggested that CT scans might help in diagnosing DMPM and other peritoneal surface malignancies (PSM) (12, 13). A meta-analysis showed that the CT scan tended to under-value the disease burden, regarding the small-volume diseases of the small intestine; these results were similar to the imaging analyses of the peritoneal diseases (14–16). However, these results might not indicate a restriction of CT scans in determining the surgical resectability of the preoperative workup for DMPM, given that high PCI is not one of the exclusion criteria for the surgical treatment of DMPM.

#### Magnetic resonance imaging (MRI)

MRI can be used as an imaging method for the diagnosis and preoperative evaluation of DMPM. It can more accurately assess the tumor progression, quantify PCI scores, diagnose peritoneal effusion, and determine disease stage (17, 18).

#### Fluorine-18 fluorodeoxyglucose (18F-FDG)-positron emission tomography-contrast-enhanced CT (PET/CT)

PET/CT has been recently introduced for the diagnosis of DMPM and has shown a promising potential due to the significant differences in the standardized uptake value (SUV) of 18F-FDG. PET/CT can be used to differentiate benign peritoneal lesions from malignant mesothelioma. Additionally, PET/CT can more accurately determine the preoperative staging lymph node status as compared to the CT alone and can also more sensitively detect the potential recurrent lesions with specificity accuracy and sensitivity of 89%, 87%, and 86%, respectively (19). These data, although heartening, require further verification by additional studies to highlight the importance of PET/CT in the preoperative evaluation of DMPM.

#### Laparoscopy

Laparoscopy is an effective method used for the diagnosis of DMPM due to its minimal invasiveness and clear visualization of the abdominal cavity. Laparoscopy can more accurately assess the resectability of DMPM, avoid ineffective open surgery, and has lower complications and mortality. Laparoscopy can better assess the small peritoneal metastatic lesions as compared to CT. The sensitivity, specificity, positive prediction value, negative prediction value, and accuracy of the laparoscopic preoperative evaluation are 100%, 75%, 96.6%, 100%, and 96.9%, respectively (20). However, for patients with poor abdominal conditions, such as previous surgery or high tumor load, laparoscopy might not achieve a comprehensive preoperative evaluation. The laparoscopic incision has a risk of implanting metastases as well (21, 22). The preoperative laparoscopy should be performed during subsequent surgery for the prevention of port site recurrence, thorough assessment of the abdominal cavity, and evaluation of serum, mesentery, and PCI (23). The biopsy of the diaphragmatic peritoneum is associated with local inflammatory reactions and adhesions, which limit the subsequent diaphragmatic peritoneal resection; therefore, surgery should be performed with caution or even avoided. The procedure of laparoscopy can be videotaped for repeated evaluation by the subsequent specialist (20).

### Diagnostic histopathology

Most DMPM cases can be easily detected or strongly suspected based on immunohistochemical (IHC) staining and routine hematoxylin and eosin (H&E) staining. The results of H&E staining for the detection of DMPM can be classified as micropapillary clear cell, tubular papillary, solid, mucinous, pleomorphic sarcomatous, and biphasic. The sarcomatous type is characterized by the presence of closely spaced spindle-shaped cells. Moreover, few sarcomatous mesotheliomas are also observed with scattered malignant bony, muscle-like, or cartilage-like structures. The biphasic type includes both the sarcomatous and epithelial subtypes and accounts for at least 10% of the DMPM cases (24). In clinical practice, IHC staining is indispensable for the pathological diagnosis of DMPM. The histological diagnosis of DMPM should be performed by an expert pathologist, because the treatment recommendations are based on the specific assessment of histological subtypes and aggressiveness, including high Ki-67 index and high mitotic rate (25). DMPM can be differentiated from adenocarcinoma and peritoneal plasmacytoma based on the IHC analysis and specific biomarkers. The IHC markers include positive markers, such as WT1(tumor suppressor gene), calretinin, and D2–40, which confirm the presence of mesothelial differentiation, and negative markers, such as carcinoembryonic antigen (CEA), thyroid transcription factor 1 (TTF-1), claudin-4, and polyclonal which confirm the presence of DMPM (26–28). Notably, no single IHC marker is completely sensitive and specific. Therefore, a combination of the positive and negative markers, including at least two mesothelial cell markers (D2-40, calretinin, WT1) and two cancer cell markers (TTF-1, CEA, polyclonal, claudin-4), is recommended for the diagnosis of DMPM (29). The most sensitive marker for sarcomatous mesothelioma is D2-40/Podoplanin (transmembrane mucoprotein) (30). The broad-spectrum keratins, such as MNF116 (pan-Cytokeratin antibody), AE1/AE3 (pan-Cytokeratin antibody), and pan-cytokeratin, are expressed in both mesothelioma and carcinoma and are not specific.

## Treatment options

DMPM was once considered an end-stage disease with a median overall survival (OS) of only 6 to 12 months after diagnosis. Recently advancements have been made in the treatment of DMPM, including both single chemotherapy and multiple forms of combination therapies, such as a combination of cytoreductive surgery (CRS) and hyperthermic intraperitoneal chemotherapy (HIPEC), systemic chemotherapy, peritoneal chemotherapy, immunotherapy, and targeted molecular therapy.

### Combination of CRS and HIPEC

**Figure 2:**
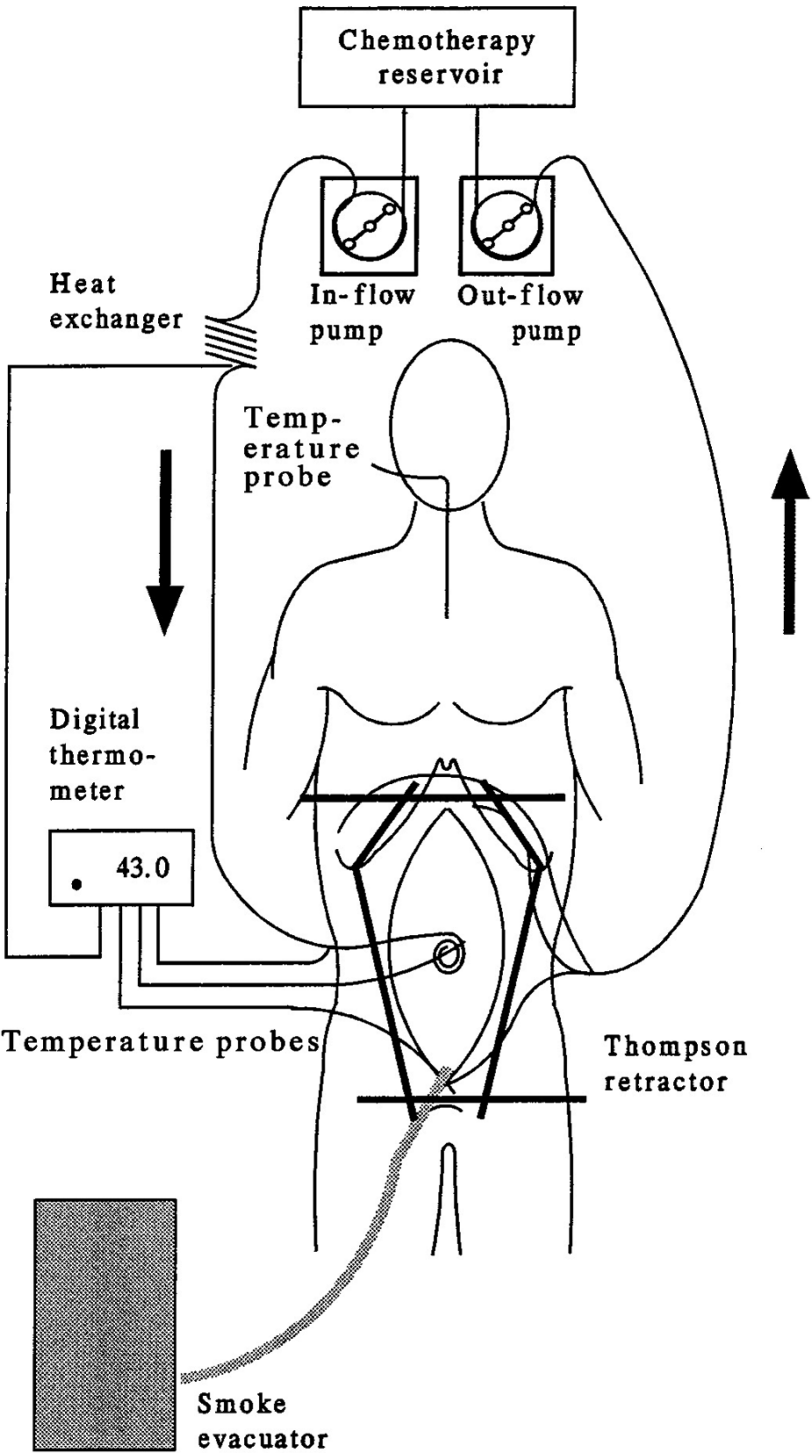
Equipment required for hyperthermic intraperitoneal chemotherapy (HIPEC) (9).

DMPM is mostly disseminated in the abdominal cavity. PSOGI recently established a comprehensive treatment strategy by combining the CRS and HIPEC as its core treatments for the resection and control of tumor progression; this strategy is preferred for the treatment of DMPM. With a median OS of 38.4 to 63.2 months, a five-year survival rate of 39.0% to 91.3%, and a perioperative mortality rate of 0 to 6%, the death risk among the patients with serious adverse events in the perioperative period is more than twice that of the patients without serious adverse events. Effective management, including preoperative, intraoperative, and postoperative management, in the perioperative period for the combined CRS and HIPEC might effectively reduce perioperative mortality (Table 2) (31–33). The combined CRS and HIPEC treatment strategy can completely resect the visible tumor, which can be seen with the naked eye. The supplementation of high-dose HIPEC can enhance treatment efficacy under hyperthermia. The most effective HIPEC regimen is the platinum-based combination of HIPEC with high-dose chemotherapeutic agents, circulating in all the regions of abdominal and pelvic cavities, under sustained hyperthermia (43°C), which enhances the cytotoxicity of the chemotherapeutic agents (Figure 2). The adverse events of combined CRS and HIPEC mainly include pulmonary infection, biliary leakage, abdominal abscess, deep vein thrombosis, anastomotic leakage, congestive heart failure, intestinal leakage, intestinal obstruction, incision dehiscence, hematological toxicity, cerebral infarction, pleural effusion, and moderate to severe hypoalbuminemia. These adverse events are correlated with the duration of surgery, PCI score, number of anastomoses, and organs or peritoneum resected (33). The adverse events are graded based on the PSOGI study (6) and consist of 48 adverse events, which are divided into 9 categories; each of which is classified as grade I-V. Grade I adverse events do not require intervention; grade II adverse events require drug therapy; grade III adverse events can be cured by conservative treatment and usually require intervention by auxiliary examinations, such as imaging; grade IV adverse events require intervention in the operation theater; and grade V adverse events are the patients' deaths. Among these, grade III-V adverse events are defined as SAE (severe adverse events).

**Table 2:** Preoperative, intraoperative, and postoperative managements for the combined CRS-HIPEC treatment.

Item	Recommendation
**Preoperative management**	
Preoperative anaesthetic assessment	Preoperative anaesthetic assessment (including cardiac risk, obstructive sleep apnea and frailty screening)
Physical exercise/prehabilitation	Prehab programme of physical exercise should be indicated routinely
Preoperative optimisation	Smoking and alcohol cessation (alcohol abusers)four weeks before surgery should be indicated routinely
	Anemia identification and correction preoperatively should be indicated routinely
Nutritional screening,supplementation	Preop nutritional screening using a validated tool and measuring serum albumin should be indicated routinel
	Nutritional and protein supplementation in cases of severe malnutrition should be indicated routinely
	Oral immunonutrition could be indicated
PONV prevention	At least 2 antiemetic drugs should be indicated routinely to prevent PONV
	TIVA could be indicated to prevent PONV
Preoperative bowel preparation	MBP alone for patients undergoing CRS ± HIPEC including probable colectomy should not be indicated
	MBP alone for patients undergoing CRS ± HIPEC including probable rectal resection could be indicated
	In patients undergoing CRS ± HIPEC, oral antibiotic decontamination with or without preoperative MBP could be indicated
Preoperative fasting and carbohydrate treatment	Preoperative fasting of 6 h for solids and 2 h for liquids should be indicated routinely
	Carbohydrate loading until 2 h before induction of anaesthesia could be indicated
**Intraoperative management**	
Antimicrobial prophylaxis and skin preparation	PrAntiseptic shower, shaving and adhesive drapes could be indicated
	Ophylactic antibiotics within 1 h before incision should be indicated routinely
	Antibiotic prophylaxis during the postoperative period should not be indicated
Standard anaesthetic protocol	Cricoid pressure during rapid sequence intubation could be indicated
	Epidural analgesia should be indicated routinely
	Multimodal analgesia with one or several agents could be indicated routinely
	Protective ventilation should be indicated routinely
	Cardiac output monitoring should be indicated routinely
	Deep neuromuscular block and reversal by specific antagonists could be indicated
Intraoperative normothermia	Prevention of intraoperative hypothermia by use of active warming devices should be indicated routinely
Intraoperative normoglycaemia	Diabetes screening and intraoperative glycaemic control should be indicated routinely
Perioperative fluid management	Use of GDFT and catecholamines guided by advanced/invasive monitoring should be indicated routinely
	Substitution of losses (fluids and protein) by use of crystalloids could be indicated
	Limiting postop fluid-related weight gain is advised
Transfusion and management of coagulopathy	Restrictive transfusion should be performed routinely
	TXA alone or with cryoprecipitate could be administered
	Prothrombin complex concentrate could be administered
Early extubation	Early extubation should be done routinely
**Postoperative management**	
Nasogastric drainage	Prophylactic nasogastric drainage should not be done
Urinary indwelling catheter	Removal of urinary catheter as early as postoperative day 3 is recommended
	Removal of urinary catheter before removal of the epidural catheter could be indicated
Postoperative analgesia	Thoracic epidural analgesia containing local anaesthetics and short-acting opiates is recommended
	After TEA removal, analgesia with paracetamol (acetaminophen), NSAIDs and opioids is recommended

PONV, postoperative nausea and vomiting; TIVA, total intravenous anesthesia; MBP, mechanical bowel preparation; GDFT, goal directed fluid therapy; TXA, tranexamic acid.

### Systemic chemotherapy (SC)

#### Palliative systemic chemotherapy

Pleural mesothelioma and DMPM are two different types of tumors. The effects of chemotherapeutic agents are similar for both these tumor types. However, the clinical trials, evaluating systemic therapy for the treatment of DPMP are limited. This might be due to the less effectiveness of single-agent and combination chemotherapies against DMPM with remission rates below 15%–20%. In phase III clinical trial, Vogelzang et al. recommended the use of pemetrexed in combination with cisplatin as a first-line chemotherapy regimen for the treatment of malignant pleural mesothelioma (34, 35). Two more studies reported that pemetrexed alone or in combination with cisplatin could effectively treat DMPM (median OS rates of 8.7 months and 13.1 months, respectively) (36, 37). The results showed that pemetrexed was well-tolerated with a low incidence of grade III/IV adverse events, among which, hematologic toxic reactions (2%) and non-hematologic toxic reactions, such as dehydration (7%), nausea (5%), and vomiting (5%), were the most common. A phase II clinical trial (38) showed that the combination of pemetrexed with gemcitabine could extend the median OS of DMPM patients to 26.8 months. However, the combined treatment regimen showed a higher incidence rate of serious adverse events. This combination is the first-line chemotherapy regimen for inoperable patients. The alternative second-line regimens include the combination of cisplatin with irinotecan or gemcitabine and tremelimumab, a monoclonal antibody against cytotoxic T-lymphocyte-associated antigen 4 (CTLA4). However, the current second-line regimens have not shown any survival advantage in the relapsed or refractory cases.

#### Perioperative chemotherapy

Adjuvant chemotherapy combined with a drug regimen is recommended for DMPM patients, receiving the combination of CRS and HIPEC and having at least one poor prognostic factor, such as sarcomatous or biphasic type, involvement of lymph node, Ki-67 > 9%, PCI >17, adjuvant chemotherapy combined with a drug regimen is advised. The patients, receiving CRS + HIPEC and having a good prognosis, such as complete CRS, epithelial type, no lymph node involvement, Ki-67 ≤ 9%, PCI ≤17, require regular follow-up. It is unclear whether the patients will be benefitted from the adjuvant chemotherapy. The most preferred chemotherapy regimen is a combination of platinum and pemetrexed.

### Peritoneal chemotherapy (PC)

PC can be used to treat malignant tumors on the peritoneal surface. The administration of high-dose chemotherapeutic drugs into the peritoneal cavity can reduce their systemic adverse effects. Studies on the intraperitoneal chemotherapy for DMPM have recommended postoperative intraperitoneal chemotherapy to enhance the efficacy of CRS and HIPEC combination therapy (39). There are two types of intraperitoneal chemotherapies. For the patients with DMPM, receiving CRS and HIPEC combination therapy, local adjuvant therapy (EPIC and/or NIPEC) can be recommended in combination with systemic chemotherapy if the postoperative clinical conditions are adequate. Long-term regional chemotherapy can improve the survival rates of DMPM patients (40). However, there is no definitive intraperitoneal chemotherapy regimen. An *in-vitro* study (41) suggested that the cisplatin and gemcitabine or cisplatin and pemetrexed combination therapies were more effective as compared to the single-agent cisplatin in thoracic chemotherapy; this study can serve as a basis for further studies on the abdominal chemotherapy regimens.

### Immunotherapy

**Figure 3:**
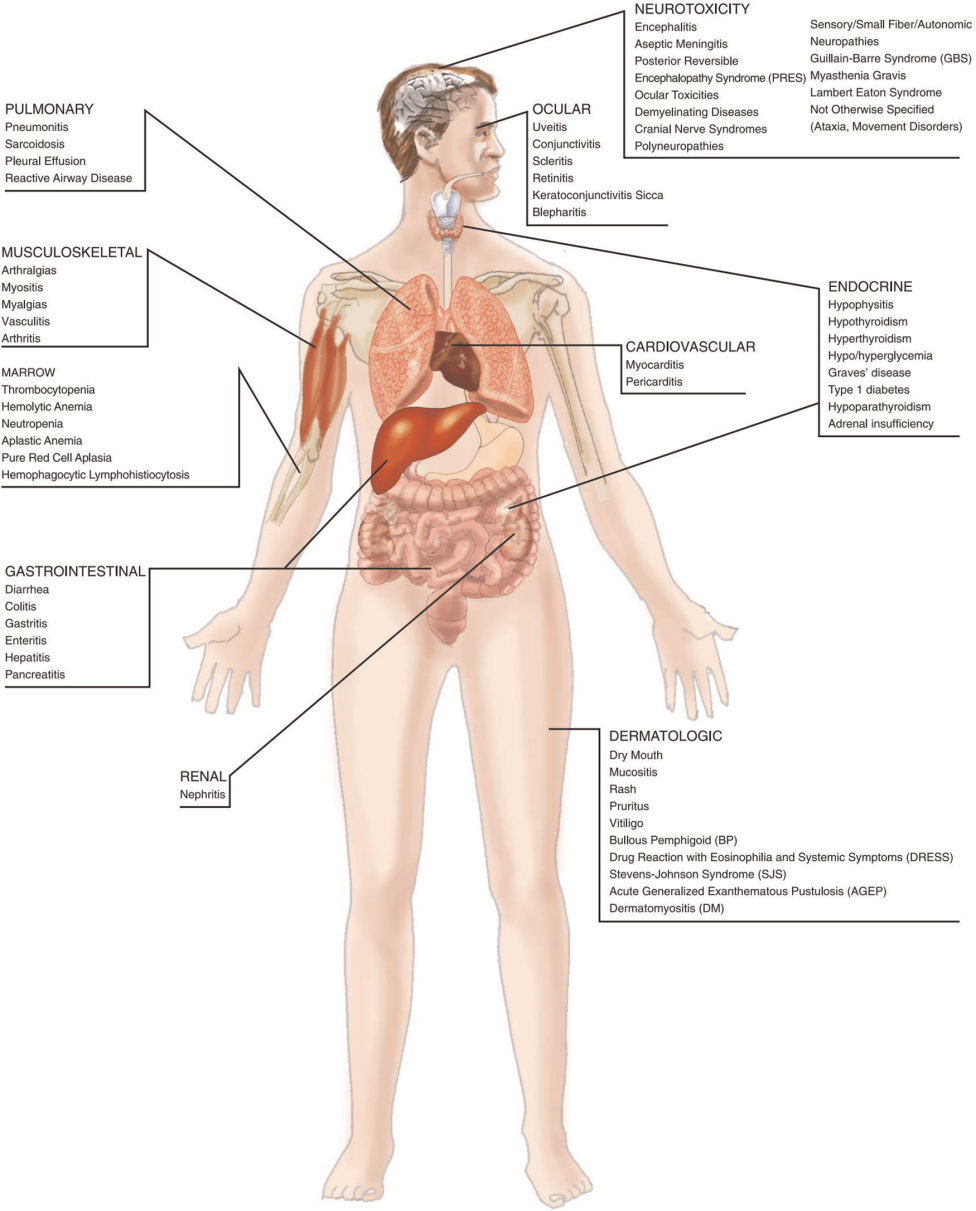
Diagram of immune checkpoint inhibitor-related toxicities (46).

Malignant mesothelioma is sensitive to immunotherapy. Currently, preclinical studies and small sample clinical trials have been conducted on immunotherapy of mesothelioma. Tumor necrosis factor-α (TNFα), interferon (IFN), granulocyte-macrophage colony-stimulating factor (GM-CSF), and interleukin-6 (IL-6) are effective immunotherapeutic agents for mesothelioma (42). Tani et al. (43) also reported that the combination of activated cytotoxic T lymphocytes (CTL) and chemotherapy was effective for DMPM patients. A phase II clinical trial (44) used tremelimumab, an anti-CTLA4 antibody, as a second-line treatment for mesothelioma, showing a disease control rate of 31% and progression-free survival (PFS) of 6 months. In addition, an animal study (45) showed that the pulse-treated dendritic cells could inhibit mesothelioma growth and control the local recurrence of mesothelioma. The immune-related drugs can kill tumor cells by blocking the negative costimulatory signaling pathways and activating the effector T cells. Simultaneously, the activated T cells can attack normal tissues and induce inflammatory cascades or even inflammatory storms by releasing cytokines, such as ILs and IFNs, resulting in various degrees of immunotherapy-related adverse reactions (irAEs). The irAEs can spread to various organ systems throughout the body, causing numerous toxicities, such as immunotherapy-related skin toxicity, gastrointestinal toxicity, liver toxicity, endocrine adverse reactions, pulmonary toxicity, bone and muscle toxicity, and rare immunotherapy-related toxicities, including neurotoxicity, cardiotoxicity, ocular toxicity, and nephrotoxicity (Figure 3) (46). The diagnosis and treatment of malignancy by a multidisciplinary team (MDT) approach through multidimensional discussions and analyses of irAEs can diagnose malignancy as early as possible, formulate a reasonable diagnosis, develop a reasonable treatment pathway and strategy, improve the efficiency of diagnosis and treatment plan, and improve the prognosis and quality of life of the patients (47). Further studies are needed to explore the efficacy of immunotherapy on DMPM.

### Targeted molecular therapy

In most DMPM patients, strong ERBB1 (HER1) activation is associated with the co-activation of ERBB2 (HER2), ERBB3 (HER3), Axl receptor tyrosine kinase (Axl), and c-Met/hepatocyte growth factor receptor (MET); this activation is mediated mainly by the heterodimerization of receptors and by an autocrine-paracrine loop, which is induced by the expression of its cognate ligand. miRNA34a can downregulate the expression of Axl (48). Mutations were found in the structural domain of MET Sema in two “progressive” DMPM patients. The combined targeted molecular therapy of Axl and MET could inhibit the cellular motility in the DMPM cell line obtained from “progressive” DMPM. A study (49) also suggested that the coordinated activity of multiple crosstalk receptor tyrosine kinases (RTKs) was directly involved in the biological processes of DMPM. These results strongly recommend that the combined inhibition of ERBB1/ERBB2 and ERBB3, MET and Axl, or PI3K/AKT/mTOR signaling pathway might be a valid therapeutic strategy, which requires further clinical investigations.

## Conclusions

DMPM is an unusual primary malignancy of the peritoneal mesothelial cell origin. The etiology and pathogenesis of DMPM are unknown. It might be caused by the interaction of carcinogenic environmental factors and the genetic susceptibility of the patients. Most early-stage patients are asymptomatic or have non-specific symptoms, thereby having a high misdiagnosis rate and poor prognosis. Some patients might benefit from the combination therapy of CRS and HIPEC. Complete CRS is an indicator of a good prognosis. The combination of pemetrexed and cisplatin is the first-line chemotherapy regimen for patients, who cannot undergo surgery. Adjuvant chemotherapy with the combination of pemetrexed and cisplatin is recommended for DMPM patients, receiving the combination of CRS and HIPEC and having at least one poor prognostic factor. The optimal outcome after combination therapy is determined by the pathological and biological markers of disease aggressiveness, such as proliferative activity and podoplanin expression. The patients, receiving the combination of CRS and HIPEC and having a favorable prognosis, require regular follow-up. Moreover, the effectiveness of adjuvant chemotherapy is needed to be further evaluated. This includes a physical examination, CT scan of the chest/abdomen/pelvis, laparoscopy, and serum cancer markers. The best practice for managing DMPM is the peritoneal surface malignancy-multidisciplinary team (PSM-MDT). PSM-MDT might significantly change the evaluation and management of DMPM. The phosphatidylinositol 3-kinase (PI3K)/AKT serine/threonine kinase 1 (AKT)/mammalian target of rapamycin (mTOR) signaling pathway is overactivated or altered in many cancer types, thereby regulating a wide range of cellular processes, such as the cellular survival, proliferation, growth, metabolism, angiogenesis, and metastasis. The overexpression of this signaling pathway also drives the malignant phenotype of DMPM, showing promising potential for developing novel interventional strategies. Further research and understanding of the molecular biology and immunology of this disease might enhance the therapeutic strategies for the long-term survival and quality of life of DMPM patients.
